# Supplementary material for: Patterns of Intron Gain and Loss in Fungi
Source: PLoS Biol. 2004 Nov 30;2(12):e422. doi: 10.1371/journal.pbio.0020422 (PMC532390; doi:10.1371/journal.pbio.0020422)
Supplement: Table S1 — Also available at http://genes.mit.edu/NielsenEtAl/. (4.3 MB ZIP). [file pbio.0020422.st001.zip › NielsenEtAl/html/912.html]

AN1162.1.NCU06035.1.MG04436.1.FG01008.1


```
 CLUSTAL W (1.82) Multiple Sequence Alignments - Introns Inserted


Sequence 1: NCU06035.1	231 aa
Sequence 2: FG01008.1	234 aa
Sequence 3: MG04436.1	229 aa
Sequence 4: AN1162.1	228 aa
Alignment Length: 235 aa
Number Identitical Residues: 108 aa
Alignment Score (without introns) 5808


MG04436.1 	MGFADILTDAGLAM1LDSWLSTRSYIVG2NSASQADVVTYKALSSAPDATKYANAARWYK
NCU06035.1	MGFTDLLTDAGLAV1LNNWLLTRSYVTG2YTASQADVAVFKALKEAPSAEKYPNAARWYK
FG01008.1 	MGFTDLLSETGAHV1LNSWLSTRSYIVG2QSASQADVAAFKALSGPPSGESYPHAARWYK
AN1162.1  	MGFTDFVSDAGLTI1ANTFFGSRSYVVG2TSPSQADVVTFKAFSSAPDAEKYPNVARWYK
          	***:*:::::*  :  :.:: :***:.*  :.*****..:**:. .*.. .*.:.*****

MG04436.1 	HIASFEDEFSNLPGDSTKAYTEYGPDKTEATLNPAKAPAAE-----EDDDDVDLFGSDDE
NCU06035.1	HIATYEDEFATLAGDSSAPYTTYGPDVAEVTINPAKAPEAAAE---EEEEDVDLFGSDDE
FG01008.1 	HIASYESQFATLPGDASAPYSTYGPGSAEVTLNPAKAPEKAEEAAAEEDDDVDLFGSDDE
AN1162.1  	HIASYEPEFGSLPGDASKEFTAYGPEGAELPTNPKDKPAAE------DDDDMDLFGSD-E
          	***::* :*..*.**::  :: ***  :* . ** . *         :::*:****** *

MG04436.1 	EEDAEAARVRQERLDEYNKKKAAK-PKTIAKSVVTLDIKPWD1DETNMVELEAAVRAIEQ
NCU06035.1	EEDAEAARIREERLAAYREKKAAK-PKIAAKSIVTMDVKPWD1DETDMVALEAAVRGIEK
FG01008.1 	EEDAEAARVREERLAEYRKKKENK-PKTIAKSVVTLDVKPWD1DETDMAALEAAVRSIEK
AN1162.1  	EEDEEVAKKHAANLAAYKAKKEAKGPKPAAKSIVTLEVKPWD1DETNLQEMEANVRAIEK
          	*** *.*: :  .*  *. **  *.**  ***:**:::**** ***::  :** **.**:

MG04436.1 	DGLVWGASKLVPVGYGVSKLQMTLVVEDDKVGVTELQEQLEELEDYVQSTDV~AAMQKL
NCU06035.1	DGLVWGGSKLVPVGFGIKKLQINMVIEDEKISLDELQEEIAGFEDYVQSTDI~AAMQKL
FG01008.1 	DGLTWGASKLVPVGFGVKKLQINLVVEDEKISVADLEEEIQEFEDYVQSTDV~AAMQKL
AN1162.1  	DGLVWGASKFVAVGFGIKKLQINMVVEDEKISIDELQAQIEEDEDHVQSTDV0AAMQKL
          	***.**.**:*.**:*:.***:.:*:**:*:.: :*: ::   **:*****: ******
```
